# Investigation on the Implementation of Mechanical Prophylaxis Procedures for Deep Venous Thrombosis in ICU in Southwest China: A Cross‐Sectional Study

**DOI:** 10.1111/crj.70069

**Published:** 2025-03-21

**Authors:** Na Li, Zhihong Tang, Yongming Tian, Xia Li

**Affiliations:** ^1^ Department of Critical Care Medicine, West China Hospital/West China School of Nursing Sichuan University Chengdu Sichuan China

**Keywords:** ICU, deep venous thrombosis, mechanical prophylaxis, implementation status

## Abstract

**Introduction:**

For ICU patients at high risk of bleeding or those already bleeding, it is recommended to use mechanical prophylaxis methods such as intermittent pneumatic compression (IPC), graduated compression stockings (GCS), or a venous foot pump (VFP).

**Objective:**

This work aims to examine the implementation of mechanical prophylaxis measures for DVT in ICUs in Southwest China and provide a foundation for improving their adoption and effectiveness.

**Method:**

In this study, a questionnaire developed by the researchers, based on existing literature, was used as the data collection tool. Following ethical approval, data were collected through self‐administered questionnaires from 780 ICU nurses across 124 ICUs in Southwest China, between August and December 2022. Of these, 67.7% (84/124) were from Grade III hospitals, and 32.3% (40/124) were from Grade II hospitals. Additionally, 66.5% (519/780) of nurses had received training on DVT prophylaxis knowledge, whereas 33.5% (261/780) had not. The data were analyzed using the Statistical Package for the Social Sciences (SPSS) software, version 21.0, with descriptive statistics and Pearson chi‐square tests applied for analysis.

**Results:**

Statistically significant differences were observed among hospitals of different grades in several aspects, including the professional management team, dynamic assessments, risk assessment records, bedside warning signs, and implement sign‐in communication for high‐risk patients (*p* < 0.05). Statistically significant differences were also found between nurses who had received training on DVT prevention and those who had not, in terms of excluding related contraindications, conducting monthly inspections and preventive maintenance, having a specially assigned person for management, and providing clear precautions (*p* < 0.05). All ICUs were equipped with at least one type of mechanical prophylaxis equipment, but the proportion and duration of equipment use varied between hospitals. The top three factors hindering the implementation of mechanical prophylaxis were insufficient equipment, inadequate human resources, and failure to reset equipment in a timely manner after disuse.

**Conclusion:**

Hospital grade, DVT prevention training, resource allocation for mechanical prophylaxis, and the implementation of prophylactic measures all influence the management of DVT mechanical prophylaxis in ICU patients. Moving forward, personalized DVT mechanical prophylaxis strategies should be tailored to the specific characteristics and needs of hospitals at different levels, with a focus on strengthening the establishment of systems, enhancing nurse training, improving equipment availability, and increasing equipment usage duration to improve the overall effectiveness of DVT prevention management.

## Graphical abstract

The equipment of mechanical prevention in ICU was insufficient. The frequency and duration of mechanical prevention measures needed to be strengthened and standardized. At the same time, the training of mechanical prevention for ICU nurses should be strengthened to improve their prevention skills and promote the implementation of nursing behaviors.

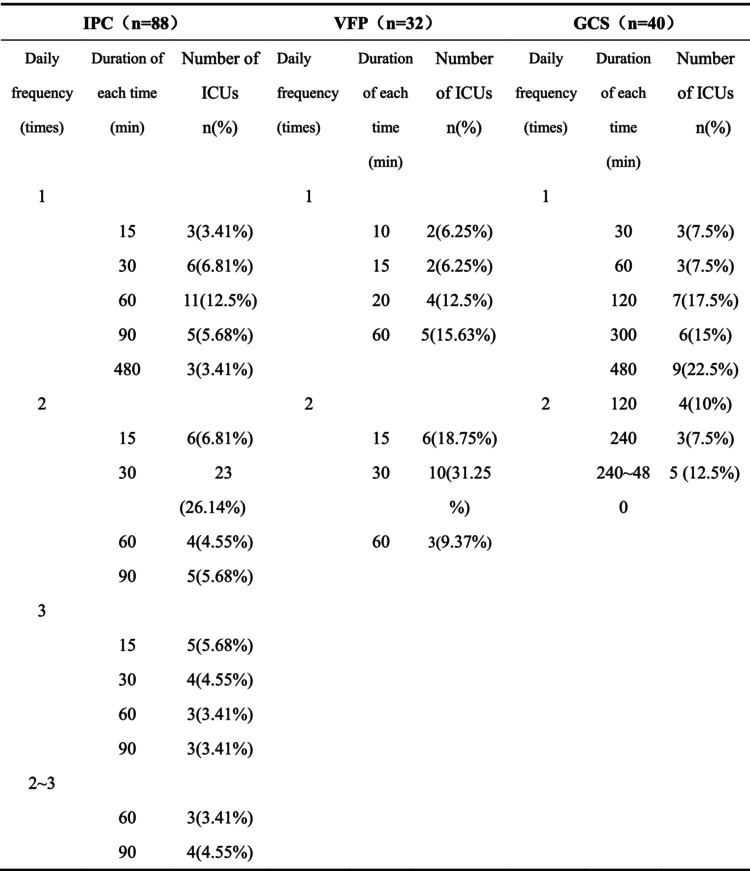

## 1 Introduction

Deep vein thrombosis (DVT), along with pulmonary embolism (PE), are leading causes of venous thromboembolism (VTE) [1]. ICU patients face a significantly higher risk of DVT because of factors such as sedative use, prolonged immobility, and trauma [1]. While the incidence of DVT in general inpatients is around 0.51% [2], ICU patients experience much higher rates, ranging from 5% to 31% [3, 4]. DVT not only leads to prolonged hospitalization and increased medical costs but can also be life threatening [5, 6]. Preventing DVT is therefore crucial for ICU patient recovery. Currently, DVT prevention methods include basic prophylaxis, drug prophylaxis, and mechanical prophylaxis. Although drug prophylaxis is effective, its anticoagulant properties increase the risk of bleeding, limiting its use [7]. In contrast, mechanical prophylaxis, including devices such as IPC, GCS, and VFP, is recommended for high‐risk bleeding patients and has proven effective in reducing DVT incidence, bleeding risks, and medical costs [8, 9, 10, 11].

ICU nurses play a pivotal role in implementing DVT prevention measures, particularly mechanical prophylaxis. Their knowledge and skills are key to the effectiveness of mechanical prophylaxis of DVT [12, 13, 14]. Nurses' current efforts in preventing DVT mainly focus on risk assessment and monitoring, but there is a need to enhance their knowledge about mechanical prophylaxis of DVT [15, 16]. A study revealed that a startling 25% of ICU medical staff have never used or even heard of mechanical thromboprophylaxis for DVT [17]. Furthermore, adequate hardware facilities and effective management systems are essential for successful implementation. Despite the importance of these factors, limited research exists on the current status of mechanical prophylaxis among ICU nurses. This cross‐sectional study in Southwest China aims to assess the management systems, practices, and existing facilities for DVT prevention in ICUs, providing insights to improve the implementation of mechanical prophylaxis among ICU nurses.

## 2 Materials and Methods

### 2.1 Research Object

In this study, ICU nurses from 124 ICUs in the southwest region of China (Sichuan Province, Chongqing, Guizhou Province, Yunnan Province, and Tibet Autonomous Region) were selected as the subjects by the convenient sampling method from August to December 2022. Inclusion criteria for hospitals was that the hospitals must be of grade II and above. Inclusion criteria for nurses was that they must be registered in‐service nurses of ICU with at least 1 year of experience in the ICU. Exclusion criteria: nurses who were unwilling to participate in the study, or sick leave, personal leave and study abroad nurses. The present study was approved by the Ethics Committee of the Biomedical Research of our hospital (Approval No.2022‐1057).

### 2.2 Research Methods

#### 2.2.1 Questionnaire Design

The questionnaire was prepared by the researcher, with reference to relevant literature [18, 19]. It was then repeatedly reviewed and modified by four ICU clinical nursing experts and two graduate nursing students. The questionnaire encompasses the hospital grade (specifically, Grade II and Grade III hospitals), nurse information (including gender, age, title, years of working experience, number of working beds, bed‐to‐nursing ratio, DVT prevention knowledge training, and training modes), the management status of the DVT hospital/department, the status of mechanical prophylaxis equipment usage for DVT (such as the use of intermittent inflation compression devices, plantar vein pumps, and thrombus elastic socks), and obstacles in the implementation of mechanical prophylaxis for DVT. Prior to the commencement of the formal survey, 20 nurses were selected to conduct a preliminary survey, which resulted in a Cronbach's α coefficient of 0.850, and the retest reliability was 0.823.

### 2.3 Method

#### 2.3.1 Research Design

A cross‐sectional study was conducted.

#### 2.3.2 Sample Size

The sample size was calculated using the following formula: *N* = *z* ^2^ *p*[(1 − *p*)/*e* ^2^], using a confidence interval (CI) of 95%, an acceptable margin of error of 5% (*e* = 5%). Based on studies estimating that 95% of ICUs in China are likely to implement mechanical prophylaxis procedures for DVT, a proportion (*p*) of 0.95 was used, resulting in a minimum sample size of 73.

#### 2.3.3 Data Collection

This research was conducted via the WeChat platform using an electronic questionnaire (accessible at https://www.wjx.cn/vm/hOrHjah.aspx). In the survey, the researcher sent the questionnaire link or QR code to the head nurse of each ICU, who then forwarded it to a randomly selected sample of nurses within their department. Each department investigated six to seven ICU nurses. After obtaining their informed consent, the researcher explained the filling method, confidentiality measures, and precautions for completing the questionnaire, emphasizing the importance of truthful answers. Nurses could access the questionnaire link or scan the QR code to answer questions online via mobile phones, computers, and other electronic devices and submit their responses directly upon completion. Invalid questionnaires (those with identical answers, missing items, obvious contradictions, or identical IP addresses for multiple submissions) were excluded, ensuring that only one valid questionnaire was retained per participant. A total of 795 questionnaires were received, of which 780 were determined to be valid, yielding an effective rate of 98.11%.

#### 2.3.4 Statistical Methods

SPSS 21.0 statistical software was used to analyze the data. The counting data were expressed in frequency and constituent ratios. The measurement data are expressed by mean value ± standard deviation, and the comparison between groups was performed by Pearson's chi‐squared test, inspection level *α* = 0.05.

## 3 Results

### 3.1 Baseline Data

The regions investigated in this study were Sichuan Province, Chongqing, Guizhou Province, Yunnan Province and the Tibet Autonomous Region. The ICUs included in the study were mainly comprehensive ICU (50, 40.3%) and Surgical ICU (31, 25.0%). The region and ICU type of the 124 ICUs are listed in Tables 1 and 2. The investigated ICU units of the hospitals (Grade III hospitals, Grade II hospitals) participating in the survey were 124; 84 (67.7%) ICU units belong to Grade III hospitals, and 40 (32.3%) ICU units belong to Grade II hospitals. The number of respondents was 780. The average number of beds in ICU of all hospitals was 26.43 ± 16.73; 30.7% of the ICUs had a bed‐to‐nursing ratio ≥ 2.5:1, with 66.5%(519/780) of nurses having received training on DVT prophylaxis knowledge; the incidence of DVT in Grade III hospitals was 20.6 ± 3.3%; the incidence of DVT in Grade II hospitals was 24.5 ± 3.9%; other basic information of respondents is listed in Supporting Information S1.

**TABLE 1:** Regions of 124 ICUs in Southwest China.

Region	ICU *n* (%)	Region	ICU *n* (%)
Sichuan Province	45 (36.3)	Yunnan Province	19 (15.3)
Chongqing	27 (21.8)	Tibet Autonomous Region	10 (8.1)
Guizhou Province	23 (18.5)		

**TABLE 2:** Type of 124 ICUs.

ICU type	ICU *n* (%)	Number of nurses *n* (%)
Comprehensive ICU	50 (40.3)	305 (39.1)
Surgical ICU	31 (25.0)	192 (24.6)
Respiratory ICU	18 (14.5)	113 (14.5)
Neurological ICU	9 (7.3)	59 (7.6)
Emergency ICU	7 (5.7)	48 (6.1)
Medicine ICU	5 (4.0)	35 (4.5)
Other ICU	4 (3.2)	28 (3.6)

### 3.2 Management Status of DVT in ICU

There were significant differences in the professional management teams, dynamic assessments, risk assessment records, bedside warning signs, and the implementation of sign‐in communication forms for high‐risk patients among different levels of hospitals (*p* < 0.05). The details indicating the management status of DVT in ICUs are listed in Table 3.

**TABLE 3:** Management status of DVT in ICU.

Projects	ICUs in Grade III hospitals (*n* = 84)	ICUs in Grade II hospitals (*n* = 40)	*χ* ^2^	*p*
Standardized prevention management system and procedures				
Yes	72	32	0.654	0.419
No	12	8		
Professional management team				
Yes	62	15	15.178	< 0.001
No	22	25		
Risk assessment for all patients				
Yes	59	21	3.724	0.054
No	25	19		
Thrombosis risk assessment scale				
Caprini	59	19	2.058	0.561
Autar	9	10		
Wells	9	6		
Other scales	6	5		
Dynamic assessment				
Yes	68	18	16.479	< 0.001
No	16	22		
Risk assessment records				
Yes	59	12	17.927	< 0.001
No	25	28		
Wrist band warning sign				
Yes	50	17	3.162	0.075
No	34	23		
Bedside warning sign				
Yes	68	12	30.729	< 0.001
No	16	28		
Implement sign‐in communication for high‐risk patients				
Yes	65	15	18.826	< 0.001
No	19	25		

### 3.3 Use Status of Mechanical Prophylaxis Equipment for DVT in ICU

There were significant differences between the trained group and the untrained group in terms of excluding related contraindications, conducting monthly inspections and preventive maintenance, having a specially assigned person for management, and providing clear precautions (*p* < 0.05). The details of the use of mechanical prophylaxis equipment for DVT in ICUs are listed in Table 4.

**TABLE 4:** The use of mechanical prophylaxis equipment for DVT in ICU (*n* = 780).

Projects	The trained group (*n* = 519)	The untrained group (*n* = 261)	*χ* ^2^	*p*
Evaluate patient compliance prior				
Yes	397	194	0.443	0.506
No	122	67		
Exclude related contraindications				
Yes	413	160	29.745	< 0.001
No	106	101		
Monthly inspections and preventive maintenance				
Yes	384	147	24.941	< 0.001
No	135	114		
Specially assigned person for management				
Yes	116	26	17.900	< 0.001
No	403	235		
Clear precautions				
Yes	423	183	12.995	< 0.001
No	96	78		
Specify the method used				
Yes	260	145	2.073	0.150
No	259	116		
Specify the mechanism of action				
Yes	289	147	0.029	0.866
No	230	114		

### 3.4 Allocation of ICU DVT Mechanical Prophylaxis Equipment

The most commonly used mechanical prophylaxis device was IPC. IPC was used in 71.0% (88/124) of the ICUs. GCS was used in 32.2% (40/124) of the ICUs. VFP was used in 25.8% (32/124) of the ICUs; 73.3% of ICUs used only one kind of mechanical prophylaxis equipment; 26.7% of ICUs used two or more types of mechanical prophylaxis equipment. The mechanical prophylaxis of DVT in ICU is listed in Table 5.

**TABLE 5:** Mechanical prophylaxis of DVT in ICU_S_ (*n* = 124).

Mechanical prophylaxis equipment	ICU_S_ (*n*)	Percentage (%)
IPC	62	50
VFP	15	12.1
GCS	14	11.2
IPC + GCS	16	12.9
IPC + VFP	7	5.7
VFP + GCS	7	5.7
IPC + GCS + VFP	3	2.4

Abbreviations: GCS: graduated compression stockings; IPC: intermittent pneumatic compression device; VFP: venous foot pump.

### 3.5 Implementation of DVT Mechanical Prophylaxis Measures by Patients

The most commonly used frequency and duration of IPC and VFP were twice a day, once for 30 min. The most commonly used frequency and duration of GCS was once a day for 480 min. The mechanical prophylaxis of DVT in ICU_S_ is listed in Table 6. The top three obstacles among factors were respectively, insufficient equipment (42.1%), inadequate human resources (25.7%) and failure to reset equipment in a timely manner after disuse (14.9%). The factors with obstacles causing mechanical prophylaxis are listed in Supporting Information S2.

**TABLE 6:** Mechanical prophylaxis of DVT in ICUs (*n* = 160).

Mechanical prophylaxis equipment	Daily frequency (times)	Duration of each time (min)	Number of ICUs *n* (%)
IPC (*n* = 88)	1	15	3 (3.4)
		30	6 (6.8)
		60	11 (12.4)
		90	5 (5.7)
		480	3 (3.4)
	2	15	6 (6.8)
		30	23 (26.1)
		60	4 (4.6)
		90	5 (5.7)
	3	15	5 (5.7)
		30	4 (4.6)
		60	3 (3.4)
		90	3 (3.4)
	2 ~ 3	60	3 (3.4)
		90	4 (4.6)
VFP (*n* = 32)	1	10	2 (6.2)
		15	2 (6.2)
		20	4 (12.5)
		60	5 (15.6)
	2	15	6 (18.8)
		30	10 (31.3)
		60	3 (9.4)
GCS (*n* = 40)	1	30	3 (7.5)
		60	3 (7.5)
		120	7 (17.5)
		300	6 (15)
		480	9 (22.5)
	2	120	4 (10)
		240	3 (7.5)
		240 ~ 480	5 (12.5)

Abbreviations: GCS: graduated compression stockings; IPC: intermittent pneumatic compression device; VFP: venous foot pump.

## 4 Discussion

DVT can lead to potentially life‐threatening complications, earning it the nickname “silent killer” [20]. Evidence suggests that early identification of high‐risk patients and appropriate prophylaxis can reduce the incidence of DVT by 50%–60% [21]. Mechanical prophylaxis, which promotes blood flow by compressing certain areas, can slow venous stasis and significantly reduce the risk of DVT [22]. Despite its proven effectiveness, clinical practice has traditionally emphasized pharmacological prevention, often overlooking mechanical prophylaxis. While healthcare professionals in China generally demonstrate a positive attitude toward mechanical prophylaxis, Xu et al. found that there is a significant gap in knowledge and standardization of practices in this area [23]. Furthermore, existing guidelines and consensus documents lack sufficient practical detail, failing to establish universally enforceable standards. In Southwest China, where healthcare systems and resources may vary significantly, it is crucial to assess the current state of mechanical prophylaxis implementation, especially in ICUs. Understanding the management systems, nurses' knowledge of mechanical prophylaxis, and the availability of necessary equipment is vital for improving DVT prevention in this region. Therefore, this study aims to explore these factors from the perspective of ICU nurses in Southwest China, providing valuable insights into the gaps and challenges in the implementation of mechanical prophylaxis procedures for DVT.

The results of this study showed statistically significant differences between Grade II and Grade III hospitals in several key areas, including the professional management team, dynamic assessments, risk assessment records, bedside warning signs, and implement sign‐in communication for high‐risk patients (*p* < 0.05). In addition, the incidence of DVT in Grade III hospitals (20.6 ± 3.3%) was significantly lower than in Grade II hospitals (24.5 ± 3.9%). This difference is likely related to the stronger organizational management capabilities in Grade III hospitals, which are reflected in more rational organizational structures, well‐established systems, and comprehensive standardized training programs. Grade III hospitals' effective thrombosis prevention leads to lower DVT incidence, whereas Grade II hospitals' higher DVT rates are partly because of lack of DVT risk awareness among management, hindering prevention protocols. Wu et al. pointed out that Grade III hospitals generally have more sufficient resources and more complete clinical pathways, allowing for more effective implementation of thrombosis prevention measures [24]. Li et al. found that Grade II hospitals, because of limited resources, often struggle to establish standardized thrombosis prevention processes, leading to a higher incidence of DVT [25]. Furthermore, some study found significant organizational management barriers in Grade II hospitals in thrombosis prevention, such as the lack of risk warning systems and dynamic risk assessments, resulting in suboptimal prevention outcomes [26, 27]. Liu et al. noted that Grade III hospitals typically have dedicated thrombosis management teams, whereas Grade II hospitals lack specialized thrombosis prevention teams, which is also one of the reasons for the higher incidence of DVT [28]. In conclusion, based on the findings of this study and other related literature, hospital managers, especially those in Grade II hospitals, should recognize their deficiencies in DVT prevention and management, prioritize the standardization of DVT prevention measures, strengthen management practices, and implement effective thrombosis prevention strategies to reduce medical risks and improve overall healthcare quality.

There were significant differences between the trained group and the untrained group in terms of excluding related contraindications, conducting monthly inspections and preventive maintenance, having a specially assigned person for management, and providing clear precautions (*p* < 0.05). These findings align with the research by Ma et al. [29], which emphasizes the crucial role of nurses in the implementation of mechanical thromboprophylaxis and the importance of strengthening their education and training in this area. Despite the recognition of the importance of proper training, clinical application of DVT mechanical prophylaxis by nurses remains inadequate, especially regarding the correct application techniques and adherence to evidence‐based guidelines [29, 30]. A growing body of literature highlights the gaps in knowledge and practice among nursing staff. For instance, a survey of 1861 healthcare professionals from 52 ICUs in northern China found that only 37.6% of respondents correctly selected prevention methods based on established guidelines [31]. This low adherence to guidelines suggests a need for more targeted and systematic training programs. Similarly, Guan et al. pointed out that the integration of guideline principles into daily practice is often inadequate in ICUs [32]. They stress the necessity for ICU nurses to receive structured, ongoing training, coupled with enhanced supervision and feedback mechanisms, to improve the effectiveness and quality of DVT mechanical prophylaxis. Moreover, these studies indicate that without proper guidance and continuous professional development, the clinical implementation of mechanical prophylaxis will remain suboptimal, potentially leading to higher rates of DVT and associated complications.

This study found that all ICUs surveyed were equipped with at least one type of mechanical thromboprophylaxis device, although the proportion and usage time varied significantly across hospitals. Among the devices evaluated, IPC, GCS, and VFP have all been shown to effectively prevent DVT when used individually [33]. Notably, 71% (88/124) of ICUs primarily used IPC, which is widely regarded as the most cost‐effective and efficient mechanical prophylaxis method. IPC not only prevents DVT but also has the potential to improve survival rates in high‐risk patients [34]. On the other hand, 32.2% of ICUs were equipped with GCS, but proper use of GCS requires careful selection of the correct size based on the patient's leg circumference and length, which may not always be feasible because of equipment limitations or inadequate measurement tools. In addition, regular monitoring of leg circumference and GCS surface integrity is essential to ensure optimal effectiveness [35]. These additional tasks can increase the nursing workload, which may contribute to lower clinical utilization of GCS. Moreover, research by Gao et al. demonstrated that combining IPC with GCS yields superior outcomes in DVT prevention compared with using GCS alone, without increasing the risk of complications. This approach reflects the growing trend of integrating multiple mechanical prophylaxis methods to enhance DVT prevention [36]. However, the current study revealed that only 26.61% (33/124) of ICUs were equipped with two or more types of mechanical prophylaxis devices, underscoring that combined use is still not universally adopted across hospitals. Furthermore, Vignon et al. found no statistically significant difference in DVT prevention between combined mechanical prophylaxis strategies and the use of GCS alone, suggesting that the clinical benefits of combining devices remain contentious [37]. Given the mixed findings in existing studies, further large‐scale, multi‐center clinical trials are needed to definitively establish the efficacy of combining different mechanical prophylaxis devices for DVT prevention.

This study revealed significant variability in the clinical use of mechanical thromboprophylaxis devices across different ICUs. Specifically, IPC and VFP were used twice a day for a maximum of 30 min, whereas GCS were used once a day for 480 min. These practices showed substantial differences in both the frequency and duration of mechanical prophylaxis, with most ICUs failing to meet the recommendations set by the American College of Chest Physicians (ACCP), which advocate for a minimum of 18 h of use per day for optimal DVT prevention [38]. In fact, the majority of ICUs in this study provided mechanical prophylaxis for less than 6 h a day, far below the expert consensus standard. Notably, no ICU reported using mechanical prophylaxis for the recommended 18 h or more, highlighting a significant gap between clinical practice and guidelines. This short‐duration use is insufficient to effectively promote blood flow and address blood supply insufficiencies, particularly in the extremities, which is crucial for preventing DVT. Several studies have shown that the limited duration of mechanical prophylaxis compromises its ability to prevent venous stasis, a key factor in DVT development [33, 34]. Moreover, inadequate usage may not achieve the sustained pressure needed to prevent venous pooling and thrombus formation. Therefore, it is essential for hospitals to prioritize DVT prophylaxis and ensure that mechanical devices are used for the full duration recommended by clinical guidelines. Only through consistent, guideline‐compliant usage can the effectiveness of mechanical thromboprophylaxis be optimized, thereby reducing the risk of DVT in critically ill patients.

This study identified the top three factors affecting the use of mechanical prophylaxis equipment in clinical practice: insufficient equipment, inadequate human resources, and failure to reset equipment in a timely manner after disuse. In line with these findings, Kinnier et al. reported that mechanical prophylaxis has the lowest compliance rate compared with drug prophylaxis [39]. A systematic review indicated that compliance with mechanical prophylaxis after surgery was approximately 75% [40]. Additionally, a prospective observational study in the United States found that 37.8% of patients were considered noncompliant because of the lack of mechanical prophylaxis equipment in the ward [41]. Ritsema et al. reported that 71.4% of patients lacked available devices on the postoperative day, and 22% still lacked devices during their subsequent hospitalization [42]. In clinical practice, it is essential to increase the availability of devices such as GCS, IPC, and VFP in proportion to the number of hospital beds to ensure efficient resource allocation. Some studies have also indicated that patient noncompliance is often because of delayed reapplication of devices after treatments, examinations, or activities [43]. According to the guidelines issued by the Critical Medicine Branch of the Chinese Medical Association (2006), the nurse‐to‐bed ratio should not be less than 1:2.543 [44]. However, this study found that only 38 ICUs (30.65%) met this standard, indicating a severe shortage of ICU nurses. This staffing shortage contributes to the insufficient mechanical prophylaxis treatment for some patients. Therefore, it is crucial to increase the allocation of nursing staff to ensure adequate care for critically ill patients. Additionally, proper maintenance and regular inspections of the equipment are essential to ensure that devices are functioning properly and to improve DVT prevention.

## 5 Limitations

This study was limited to ICUs in Southwest China, with the majority located in Grade III hospitals. As a result, the findings may not fully represent the situation in ICUs across hospitals of different grades or regions. Future research should expand to include ICUs nationwide to provide a more comprehensive understanding of the current status of DVT mechanical prophylaxis and management in hospitals of all levels.

## 6 Conclusions

In conclusion, we conducted a questionnaire survey to assess the current status of DVT mechanical prophylaxis in ICUs in Southwest China, focusing on the perspectives of ICU nurses and their implementation of mechanical prophylaxis procedures. The results revealed that improvements are needed in the management systems and nurses' knowledge of mechanical prophylaxis in Grade II hospitals, while Grade II and Grade III hospitals need to enhance the availability of necessary equipment. These findings provide valuable insights that can inform future improvement plans, ultimately enhancing the quality of DVT mechanical prophylaxis in ICUs.

## Author Contributions

Na Li contributed to the data analysis and the writing of the research manuscript; Zhihong Tang and Yongming Tian performed data collection; Xia Li performed the data analyses.

## Ethics Statement

This survey was reviewed and approved by the ethics committee of West China Hospital of Sichuan University, which waived the need for informed consent.

## Conflicts of Interest

The authors declare no conflicts of interest.

## Supporting information


**Data S1** Basic information of respondents

**Data S2** Factors with obstacles causing mechanical prevention (*n* = 780)

## Data Availability

The data that support the findings of this study are available from the corresponding author upon reasonable request.
