# Supplementary material for: Investigation on the Implementation of Mechanical Prophylaxis Procedures for Deep Venous Thrombosis in ICU in Southwest China: A Cross‐Sectional Study
Source: Clin Respir J. 2025 Mar 21;19(3):e70069. doi: 10.1111/crj.70069 (PMC11926399; doi:10.1111/crj.70069)
Supplement: Supplementary file 1 — Data S1 Basic information of respondents [file CRJ-19-e70069-s001.doc]

**Supplement 1 Basic information of respondents**

| **Projects** | **Number of nurses**  **（n=780）** | **Number of ICUs**  **( n=124）** |
| --- | --- | --- |
| **Hospital grade (case)** | | |
| Grade III hospitals | 488(62.6%) | 84(67.7%) |
| Grade II hospitals | 292(37.4%) | 40(32.3%) |
| **Average number of beds(bed)** | 26.43±16.73 | |
| **bed-to-nursing ratio** |  | |
| ≥2.5:1 | 230(29.5%) | 38(30.7%) |
| <2.5:1 | 550 (70.5%) | 86(69.3%) |
| **Gender（case）** | | |
| Male | 132(16.9%) |  |
| Female | 648(83.1%) |  |
| **Age（year）** | 35.93±6.25 | |
| **Title** | | |
| Nurse | 131 (16.8%) |  |
| Nurse-in-charge | 462 (59.2%) |  |
| Co-chief superintendent nurse | 99 (12.7%) |  |
| Chief superintendent nurse | 88 (11.3%) |  |
| **Working years (year)** | 7.98±4.87 | |
| **DVT prevention knowledge training (case)** |  | |
| Yes | 519 (66.5 %) |  |
| No | 261 (33.5%) |  |
| **Training frequency of VTE** |  |  |
| Quarterly | 56(10.8%) |  |
| Semiannual | 191(36.8%) |  |
| Annually | 272(52.4%) |  |
| **Training modes (case)** |  | |
| Face to face | 277 (53.4%) |  |
| Workshop | 189 (36.4%) |  |
| Online | 53 (10.2%) |  |
| **Incidence of DVT(%)** |  |  |
| Grade III hospitals | 20.6±3.3 |  |
| Grade II hospitals | 24.5±3.9 |  |
