# Supplementary material for: Investigation on the Implementation of Mechanical Prophylaxis Procedures for Deep Venous Thrombosis in ICU in Southwest China: A Cross‐Sectional Study
Source: Clin Respir J. 2025 Mar 21;19(3):e70069. doi: 10.1111/crj.70069 (PMC11926399; doi:10.1111/crj.70069)
Supplement: Supplementary file 2 — Data S2 Factors with obstacles causing mechanical prevention (n = 780) [file CRJ-19-e70069-s002.doc]

**Supplement 2 Factors with obstacles causing mechanical prevention (n=780)**

| **Projects** | **Cases（n=780）** | **Proportion（%）** |
| --- | --- | --- |
| Insufficient equipment | 328 | 42.1 |
| Insufficient human resources | 201 | 25.7 |
| Ailure to reset equipment in a timely manner after disuse | 116 | 14.9 |
| Financial burden of patients | 47 | 6.0 |
| Difficulty in putting on and off | 42 | 5.4 |
| Handover skin problems | 31 | 4.0 |
| Other issues | 15 | 1.9 |
